# Improving Beef Cattle Production: Safety and Effectiveness of New Immunocastration Vaccine

**DOI:** 10.3390/ani14172538

**Published:** 2024-08-31

**Authors:** Daniela Siel, Paula R. Huenchullán, Sonia Vidal, Andrea Valdés, Leonardo Sáenz

**Affiliations:** 1 Laboratory of Veterinary Vaccines, Department of Animal Biology, Faculty of Veterinary and Animal Science, Universidad de Chile, Santiago 8820808, Chile; daniela.siel@umayor.cl (D.S.); soniavidal@uchile.cl (S.V.); a.valdesaguilera@gmail.com (A.V.); 2 Escuela de Medicina Veterinaria, Facultad de Medicina y Ciencias de la Salud, Universidad Mayor, Santiago 8580745, Chile; 3 Centro de Biomedicina, Universidad Mayor, Santiago 8580745, Chile; 4 Magister Bienestar Animal y Etología Aplicada, Universidad de las Américas, Providencia 7500975, Chile; pramirezh@udla.cl

**Keywords:** immunocastration, GnRH, beef cattle, animal welfare

## Abstract

**Simple Summary:**

The research focuses on the development and evaluation of a new immunocastration vaccine for beef cattle. Controlled and on-farm trials were conducted to evaluate safety, immunogenicity, immunocastration efficacy, and production parameters. The vaccine demonstrated safety and efficacy in inducing specific anti-GnRH antibodies, reducing estrus presentation and progesterone concentration, and improving production parameters. This research presents promising results for the efficacy and safety of the immunocastration vaccine, highlighting the potential benefits for reproductive management of beef cattle.

**Abstract:**

Reproductive control in mammals, particularly in beef production systems, is crucial for managing behaviors driven by sex steroids, which can cause biosecurity issues, reduced weight gain, and decreased meat quality. Additionally, controlling sexual activity in females prevents unwanted pregnancies when both sexes are housed together for fattening. Surgical castration in cattle, commonly performed under field conditions, is associated with significant welfare concerns due to pain and infection risks. Furthermore, castrating female cattle in the field is often impractically expensive. Hence, new reproductive control methods that prioritize animal welfare and are suitable for both sexes are essential. Immunocastration (IC), which involves vaccination against the GnRH-I hormone, has emerged as a promising alternative, demonstrating efficacy and safety in various species. Immunocastration has proven to be an effective alternative to surgical castration in controlling reproduction and promoting animal welfare in several species. This study aims to evaluate the safety, immunogenicity, immunocastration effect, and productive impact of a novel IC vaccine developed in Chile for female cattle. Two consecutive trials were conducted, the first under controlled conditions and the second under field conditions. The results demonstrated that the new vaccine is safe and effective for use in beef cattle, inducing specific immunity against GnRH-I, reducing gonadal functionality, and improving productive parameters. These findings suggest that this new IC vaccine can significantly benefit the beef cattle industry by providing a welfare-friendly and efficient method of reproductive control.

## 1. Introduction

Reproductive control in mammals has been a topic of interest for several decades. In beef animal production systems, reproductive control is needed to control agonistic or other undesirable behaviors dependent on sex steroids, which lead to biosecurity problems and reduced weight gain and meat quality [1,2,3], as well as inhibiting sexual activity in females which can result in unwanted pregnancies when females and males are housed together for fattening [4,5,6,7].

In cattle, surgical castration is often performed under field conditions, which is associated with pain, risk of infection, and compromised animal welfare. Castration of female cattle in the field is nearly impossible as the cost is too high. Therefore, it is necessary to develop new methods of reproductive control that go hand in hand with animal welfare, do not cause unnecessary pain, and are applicable to both sexes in different species in order to meet the demands of modern society [8,9]. 

Gonadotropin-releasing hormone (GnRH-I) is a hypothalamic decapeptide that triggers the release of follicle-stimulating hormone (FSH) and luteinizing hormone (LH). This, in turn, stimulates the secretion of gonadal steroids and the development of gametes [10,11].

Immunocastration (IC), or vaccination against the GnRH-I hormone, has emerged as a promising tool for reproductive control in mammals, as it has been shown to be effective and safe in different species [3,12,13,14], such as dogs [15] and pigs [16,17], for controlling reproduction and improving animal behavior and welfare.

Some studies have demonstrated the efficacy of IC in inducing specific anti-GnRH antibodies in females, decreasing ovarian function and progesterone concentration, preventing follicular growth, and inducing anestrus [5,18]. In females of productive species, the main objectives of blocking reproductive activity are to control sexual behavior, avoid unwanted pregnancies, and prevent losses that occur during estrus due to decreased feed intake and daily weight gain [5,19,20,21]. Currently, evidence concerning the efficacy of IC in female cattle is scarce and poorly updated [5,19,22,23]. However, in pigs, immunocastration appears to be increasing the homogeneity between sexes (males and females) in terms of productive performance and quality [24].

Regarding animal welfare, castration of female cattle is desirable when they are not going to be used for reproduction. When females enter estrus, they begin to have altered behavior, which generates restless and stressed herds, a situation that is avoided by castrating them [25].

In this study, two consecutive trials in female cattle were performed, the first under controlled conditions and the second under field conditions, with the objective of evaluating the safety, immunogenicity, immunocastration effect, and productive effect of a new vaccine for immunocastration of cattle developed in Chile.

## 2. Materials and Methods 

The experiments were carried out in the Porcine Animal Management Unit at the Faculty of Veterinary and Animal Sciences at the University of Chile (Metropolitan Region of Santiago, Chile). All experiments were approved by the Institutional Animal Care and Use Committee of the University of Chile (Certificate No. 22552-VET-UCH-e1) and adhered to the ARRIVE guidelines [18].

### 2.1. Vaccine Formulation

The vaccine used was the GnRXG/Q vaccine, a recombinant vaccine developed at the Veterinary Vaccine Laboratory of the University of Chile [3,14,15,26,27]. Each dose (2 mL) of the prepared vaccine contained 1 mg recombinant GnRXG/Q protein, 10 mg soluble low-molecular-weight chitosan (Sigma Aldrich, St. Louis, MO, USA), and NaCl 0.9% as excipient. Placebo groups were vaccinated with adjuvant alone in the absence of antigen. Vaccines were administered subcutaneously on days 1 and 21 in the neck using 5 mL syringes and 18 G needles. Each animal received two doses of vaccination, which were performed in the same area in an adjacent manner in an anteroposterior direction, at a distance of approximately 2–3 cm, to easily monitor the evolution of the injection site.

Two consecutive studies were conducted to evaluate the safety and efficacy of the new immunocastration vaccine. The first was a safety and efficacy study under controlled conditions and the second was under field conditions.

### 2.2. Controlled Conditions Study

Twenty-five crossbred heifers between 14 and 15 months of age with an average weight of approximately 250 kg were used. The animals were randomly divided into 5 groups of 5 animals each, identified by an ear tag in the right auricle. The identification number was recorded for each animal according to the official registration number of the Agriculture and Livestock Service (SAG). Only clinically healthy animals without clinical signs of pathology (normal coat, stool, and behavior) were included in the study.

Animals were dewormed internally and externally prior to the study. Prior to the start of the study (first vaccination), the animals were acclimatized for approximately one month. The cattle were kept under permanent confinement, housed in a common pen of approximately 1600 m^2^. The ambient temperature, ventilation, and light were not artificially controlled, so the ambient temperature varied between 10 and 30 °C and the light/dark ratio was approximately 12/12. The test animals’ diet consisted of alfalfa hay at a rate of six bales per animal per day, divided into two daily rations. Drinking water was potable water administered as needed.

At the end of the study, the animals were made available to the animal production department of the Faculty of Veterinary and Livestock Sciences of the University of Chile for teaching purposes.

### 2.3. Field Test Conditions

Ninety-nine whole female cattle, 14 to 15 weeks old (heifers), were randomly assigned to 2 groups and individually identified by the official registration number of the Agriculture and Livestock Service (SAG). One group was vaccinated with the vaccine antigen (n = 79) and the placebo group (n = 20) was vaccinated with chitosan only.

Only healthy females with no visible changes on visual inspection, no history of reproductive pathology or abortion, and with a body condition of 3.25–3.50 were included. The animals under study were subjected to a monitoring protocol that allowed the assessment of the physiological status of the animal and the possible occurrence of adverse reactions using the score previously described in Section 2.4. 

Any animal scoring 3 or more points was immediately removed from the study and appropriate palliative and management measures were taken.

Animals were housed under real field conditions in a farm located in the Metropolitan Region of Santiago, Chile. Feeding and care of the animals were carried out in separate pens per group and were the responsibility of the farm personnel, who were previously trained. A veterinarian expert in bovine medicine participated as an observer of the study. At the end of the study, the animals were slaughtered according to the regulations of the farm.

### 2.4. Vaccine Safety Analysis

After each immunization in the control and field studies, the animals were observed for 5 consecutive days to assess the occurrence of local and/or systemic signs or symptoms. By visual inspection, the local reaction was graded as follows: 0: no reaction; 1: visible inflammation but not abscessed; 2: non-ulcerated abscess; 3: ulcerated abscess; 4: signs of systemic hypersensitivity.

To evaluate possible systemic effects of vaccination, we performed visual observation of behavior, posture, and general condition of the animals, as well as biochemical profile and hemogram before and at the end of the study. For the safety assessment, we used the score developed in bulls in our previous study [3].

### 2.5. Analysis of Vaccine Efficacy

#### 2.5.1. Collection of Blood Samples

For the evaluation of serum levels of progesterone and specific immunoglobulins in both studies, blood samples were collected individually on days 1, 21, and 64. Blood samples were collected by puncture of the caudal vessels in vacuum blood collection tubes without anticoagulant. The samples were centrifuged at 2000× *g* for 10 min, and the collected serum was stored at −20 °C until used for specific assays. The samples were analyzed according to company regulations. 

#### 2.5.2. Measurement of Specific Antibodies in Blood

The immune response induced by vaccination was determined by measuring specific antibodies (IgG) to GnRH-I hormone throughout the evaluation period. Measurement of specific antibody levels was performed by indirect enzyme-linked immunosorbent assay (ELISA). Briefly, the assay was performed in 96-well plates (Nunc^®^ MaxiSorp, 240421, San Diego, CA, USA) in which 2 μg GnRH-I (Sigma Aldrich) was bound with 50 μL binding buffer (Na_2_CO_3_ 150 mM; NaHCO_3_ 350 mM; pH 9.6) for 18 h at 4 °C. The plates were then washed twice with wash buffer (Tween 20 0.05% *v*/*v*; PBS) and blocked with 200 μL blocking buffer (5% *w*/*v* nonfat milk; PBS) for 2 h at 37 °C. The plates were then washed twice with wash buffer and incubated for 2 h at 37 °C with 100 μL of each serum diluted 1:100 in diluent buffer (0.5% *w*/*v* nonfat milk; PBS). After incubation, the plates were washed five times and incubated with 100 μL of peroxidase-conjugated polyclonal anti-bovine IgG antibody (Jackson Immunoresearch Laboratories, Inc., West Grove, PA, USA), diluted 1:10,000 in dilution buffer, for 1 h at 37 °C. Finally, the plates were washed 5 times and developed with 100 μL 1-Step™ Ultra TMB ELISA (Pierce, Chemical Company, Jamestown, RI, USA) for 3 min at room temperature. The reaction was stopped with 100 μL stop solution (1.5 M H_2_SO_4_, in PBS) and the absorbance was read at 450 nm.

#### 2.5.3. Measurement of Progesterone Levels

In both studies, chemiluminescence was performed at the Vetlab veterinary laboratory (external laboratory) to evaluate the progesterone concentration in the serum of the study animals from blood samples collected on days 1, 21, and 64.

#### 2.5.4. Estrus Recording

In the control conditions study, a veterinarian recorded the estrus presentation of the study females for one hour each day at two different times (AM and PM), indicating the registration number of the animal and the date of estrus presentation.

#### 2.5.5. Productive Parameters

The following productive parameters were determined only in the field trial:

Feed intake (FI): FI was determined daily, using the pen as the experimental unit (vaccinated pen and unvaccinated pen), by the difference between the feed delivered and the feed remaining at the end of the day.

Dry matter intake (DMI): DMI was calculated on the basis of the FI, considering that 50.15% of the feed administered is dry matter.

Body weight (BW) and daily weight gain (DWG): Animals were weighed at the beginning of the study (day 1) and at the end of the study (day 64). DWG was calculated as the difference between day 60 and day 1 for the entire study period [(DWG day 64 − DWG day 1)/64 days].

Feed Conversion Efficiency (FCE): FCE was calculated using the pen as the experimental unit. DMI and daily weight gain (DMI/GW) were used for the calculation.

### 2.6. Statistical Analysis

Comparisons between groups were performed using the non-parametric Kruskal–Wallis test and Dunn’s post hoc test. GraphPad Prism version 8 software was used for the analyses. The differences were regarded as significant with values of *p* < 0.05. The results are expressed as the mean ± standard deviation (SD).

## 3. Results

### 3.1. Controlled Trial

#### 3.1.1. Safety

Heifers were observed and inspected daily to assess the occurrence of local and/or systemic adverse signs. Throughout the study, the animals scored 0 for both assessments, i.e., no local or systemic adverse effects were observed.

#### 3.1.2. Specific Immunity

Indirect ELISA was used to assess the production of anti-GnRH-I IgG in the different experimental groups. 

**Figure 1:**
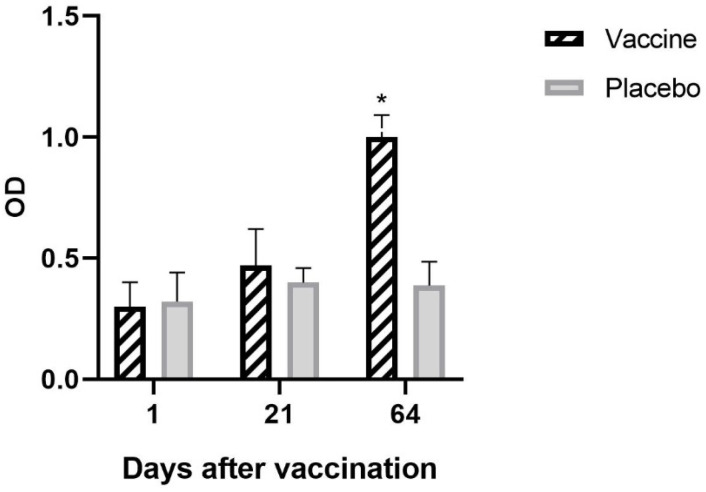
Specific anti-GnRH-I IgG antibodies in the serum of placebo and control animals in the controlled conditions study. Animals were vaccinated on days 1 and 21 of the study. Indirect ELISA was used to measure anti-GnRH-I IgG levels in the serum of vaccinated and placebo animals. A significant increase in serum IgG was observed in vaccinated animals on day 64 of the study. Data are expressed as optical density (OD) ± DS. Asterisks indicate significant differences (*p* < 0.05).

As shown in Figure 1, the vaccinated group showed an increase in anti-GnRH-I IgG levels at the end of the study (day 64), i.e., after the two doses of vaccination. In the placebo group, no increase in antibodies to the native hormone was observed (Figure 1).

#### 3.1.3. Effect on Reproductive Function

**Figure 2:**
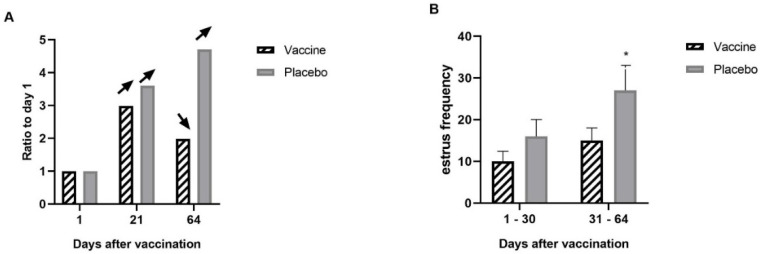
Progesterone levels and estrus in the controlled conditions study. Animals were vaccinated on days 1 and 21 of the study. (**A**) Serum progesterone was measured by chemiluminescence in vaccinated and placebo animals. An increase in progesterone was observed in both groups on day 21 compared to day 1. At day 64, an increase in serum progesterone levels was observed that continued in the placebo group. In contrast, a decrease in serum progesterone levels was observed in the vaccinated animals on day 64. Data are presented as ratios relative to day 1 (day 1 was considered as a value of 1). Arrows indicate increase or decrease relative to the previous time. (**B**) Estrus presentation was assessed by visual inspection in the vaccinated and placebo groups. Estrus presentation was determined for each group during two periods, days 1 to 30 and days 31 to 64. It was observed that only in the placebo group was there an increase in the frequency of estrus presentation during the second period. Data are presented as mean estrus frequency for each group ± DS. The asterisks indicate significant differences (*p* < 0.05).

Chemiluminescence was used to determine progesterone levels (ng/mL) in the animals studied. Since sex steroids are highly variable among individuals regardless of their reproductive status, to visualize the effect of vaccination on progesterone levels, the values obtained were plotted as a ratio based on day 1 (day 21/day 1; and day 64/day 1). In addition, day 1 was plotted with a value of 1 (day 1/day 1) to better visualize the change in progesterone ratio over time (Figure 2A).

As shown in Figure 2, on day 21 of the study, progesterone increased in both groups compared to day 1. At day 64 of the study, progesterone continued to increase in the placebo group as expected with increasing development of the animals. In contrast, a decrease in progesterone was observed in vaccinated animals compared to day 21 (Figure 2A).

The determination of estrus was divided into two periods, the first between days 1 and 30 of the study and a second between days 31 and 64. As expected with the increasing age of the heifers and in agreement with the results obtained for the hormone progesterone, estrus tended to increase in the second period of the study in all animals, but this increase was significantly higher in the animals of the placebo group (Figure 2B).

### 3.2. Field Trial

#### 3.2.1. Safety of the Vaccine 

By daily visual observation, the farm operators and the veterinarian in charge of the field confirmed that the vaccine was safe, i.e., it did not induce any local or systemic side effects, with a score of 0 for both studies in all animals.

#### 3.2.2. Specific Immunity

**Figure 3:**
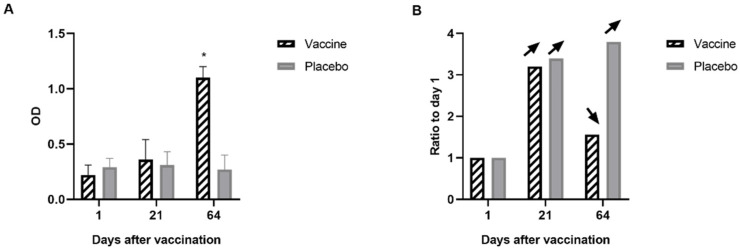
(**A**): Specific anti-GnRH-I IgG antibodies in the serum of placebo and control animals in the study under field conditions. Animals were vaccinated on days 1 and 21 of the study. An indirect ELISA was utilized to measure anti-GnRH-I IgG levels in the serum of animals from both the vaccinated and placebo groups. A significant increase in serum IgG was observed in the vaccinated animals on day 64. Data are expressed as optical density (OD) ± DS. Asterisks indicate significant differences (*p* < 0.001). (**B**): Serum progesterone was measured by chemiluminescence in animals from the vaccinated and placebo groups. An increase in progesterone levels was observed in both groups on day 21 compared to day 1. On day 64, a continuous increase in serum progesterone levels was noted in the placebo group. Conversely, the vaccinated animals exhibited a decrease in serum progesterone levels on day 64. Data are presented as ratios relative to day 1 (day 1 was considered as a value of 1). Arrows indicate an increase or decrease relative to the previous day.

In vaccinated animals, an increase in antibodies was observed from day 21 in vaccinated animals, but the increase was not statistically significant until day 60 of the study, after the second vaccination. No immune response to GnRH-I was observed in placebo animals (Figure 3A).

#### 3.2.3. Effect on Reproductive Function

As shown in Figure 3B, at day 21 of the study, progesterone increased in both groups compared to day 1, which is expected due to the sexual development of the animals. At day 64 of the study, progesterone continued to increase in the placebo group, while a decrease in progesterone was observed in the vaccinated animals compared to day 21. The second vaccination produced an immunocastration effect that inhibited the increase in sex steroids (Figure 3B).

#### 3.2.4. Assessment of Productive Parameters

Using the pen as the experimental unit, the following productive parameters were determined during the 60-day study: daily weight gain (DWG), dry matter intake (DMI) and Feed Conversion Efficiency (FCE). As shown in Table 1, vaccinated animals had a better FCE (5.58) compared to the placebo group (5.69), i.e., vaccinated animals had to consume 5.58 kg DM to produce 1 kg body weight and unvaccinated animals had to consume 5.69 kg DM to produce 1 kg body weight (Table 1).

## 4. Discussion

The control of reproductive activity in males and females is a subject of great interest to the various fields of veterinary medicine. Surgical castration methods are the most common sterilization techniques. However, these traditional methods can cause pain and infection when conducted under field conditions and are generally detrimental to animal welfare [28]. In addition, it is impractical to use traditional methods of castration on female cattle given the working conditions on the farm. As a result, beef producers face significant challenges in finding alternatives to traditional castration [29].

Immunocastration, or vaccination against the GnRH-I hormone in mammals, has emerged as a promising alternative to address these challenges, proving to be safe and effective in different species [3,12,13,14]. European countries are promoting GnRH immunocastration as an alternative to surgical castration to improve animal welfare. Immunocastration reduces animal stress, reduces the risk of infection and complications associated with surgical castration, reduces pain, and improves animal welfare. GnRH immunocastration is considered a relatively safe alternative to surgical castration [16]. This technology has shown good results mainly in males [3,16,30], but its usefulness in female cattle has been little studied. Immunocastration has previously been shown to delay puberty in calves, but its subsequent effect on productive parameters has not been studied [30]. In addition, immunocastration has shown successful results in females of other productive species, such as pigs [31].

Also, the meat from immunocastrated cattle does not contain harmful residues that could pose a risk to human health. Regulatory bodies such as the European Medicines Agency (EMA) and the U.S. Food and Drug Administration (FDA) have evaluated and approved these vaccines, ensuring they meet stringent safety standards. 

In this study, two consecutive trials were conducted, one under controlled conditions and the other under field conditions, to evaluate immunogenicity and effects on reproductive and productive parameters in bovine females.

It was observed in both trials that it was possible to induce a specific immune response against the native hormone GnRH-I, thus fulfilling the first basic requirement for an effective immunocastration vaccine. Vaccination of animals produces specific antibodies against GnRH-I, which inactivates endogenous GnRH-I, thereby reducing the levels of gonadotropins and thus sex steroids such as progesterone [32]. 

Immunometrology, the study of measurement standards for immune responses, plays a crucial role in evaluating the efficacy of vaccines, including immunocastration vaccines. One key concept within this field is the law of the ebb and flow of vaccine antibody levels, which describes the dynamic changes in antibody concentrations post-vaccination. After an initial peak following vaccination, antibody levels typically decline over time, reaching a plateau. The critical point of antibody level, also known as the protective threshold, is essential to ensure the effective immune effect of immunocastration vaccines. This threshold must be maintained to prevent the physiological functions that the vaccine aims to suppress, such as reproductive hormone production in animals. Studies suggest that maintaining antibody levels above this critical point is vital for long-term efficacy and can vary depending on the vaccine formulation and the animal species involved [33]. 

In this study, the duration of efficacy of the two-dose vaccination was evaluated over 64 days. Future studies of longer duration should be developed to further characterize the immunometry associated with this new immunocastration vaccine.

Our results showed that in both controlled and field conditions, vaccination induced a decrease in progesterone relative to day 1 of each study. This was also associated with a decrease in the occurrence of estrus in vaccinated females when evaluated under controlled conditions.

Finally, the evaluation of productive parameters in the field trial showed an improvement in the vaccinated animals. This is very important because it is the first evidence that immunocastration can have a positive effect on the productive efficiency of female cattle. According to our results, a vaccinated animal needs to consume 110 g less feed to produce 1 kg of weight. Considering that an animal with these characteristics fattens to an average of 100 kg in two months, each vaccinated animal would consume approximately 11 kg less feed than a control animal to gain the same weight, which represents significant savings for the production herd. 

Although our results are promising, we believe that we can improve our methodologies in future studies. For example, in this study, immunity was expressed as gross optical density (OD). In future studies, we would consider determining antibody titers and expressing the results in that unit to achieve greater precision in the analyses. In addition, we consider it pertinent to consider other dates of antibody evaluation in search of the most optimal points for the evaluation of specific immunity [25].

Previous results from our team show that this vaccine improves meat quality in bulls. In this study, Feed Conversion Efficiency was considered as the main productive indicator. However, other parameters associated with meat quality should be considered in future studies to truly measure the economic benefit of using this technology on female cattle in particular [3].

This new vaccine for the immunocastration of cattle emerges as an alternative not only for use in male cattle [3] but also in female cattle, requiring further study for its use in beef production at national and global levels.

## 5. Conclusions

The control of reproductive activity in farm animals has been a topic of interest for decades. Some of the main problems with surgical castration are that it compromises animal health and welfare when performed under field conditions and that it is not feasible in females. In beef cattle production, the control of reproductive activity is relevant for the management of estrus and unwanted pregnancies and the improvement of production parameters. Immunocastration is emerging as an alternative to surgical castration to address this problem. 

In this study, two consecutive trials were conducted in female cattle to test a new immunocastration vaccine developed in Chile, the first under controlled conditions and the second under field conditions. In both trials, the vaccine was found to be safe and effective. The results show that immunocastration is capable of controlling reproductive functionality and improving production parameters in beef cattle, making it a promising alternative for reproductive control in the industry.

## Figures and Tables

**Table 1:** Production parameters evaluated during the 60-day field trial.

Groups	DWG (kg)	DMI/Animal (kg)	FCE
Vaccinated	1.73	10.21	5.58
Placebo	1.81	10.32	5.69

The values presented in the table are averages considering the pen as the experimental unit (pen 1: vaccinated animals; pen 2: placebo animals). DWG: daily weight gain. DMI: dry matter intake. FEC: Feed Conversion Efficiency.

## Data Availability

The data presented in this study are available on request from the corresponding author.
